# Histological and clinical evaluation of novel deproteinized porcine bone mineral in socket preservation: a case report

**DOI:** 10.3389/froh.2026.1719449

**Published:** 2026-02-13

**Authors:** Jie Chen, Lina Xu, Zixiao Wang, Qing Wang

**Affiliations:** 1 Department of Stomatology, Zhongshan Hospital, Fudan University, Shanghai, China; 2 Department of Periodontology, Shanghai Ninth People’s Hospital, Shanghai Jiao Tong University School of Medicine; College of Stomatology, Shanghai Jiao Tong University; National Center for Stomatology; National Clinical Research Center for Oral Diseases; Shanghai Key Laboratory of Stomatology, Shanghai Research Institute of Stomatology, Shanghai, China; 3 Department of Oral Implantology, Stomatological Hospital of Xuhui Distinct, Shanghai, China

**Keywords:** bone regeneration, case report, deproteinized porcine bone mineral, Masson trichrome staining, socket preservation

## Abstract

Socket preservation, also known as alveolar ridge preservation, is a crucial dental procedure aiming to minimize the resorption of the alveolar ridge following tooth extraction. The use of bone substitutes has reduced the morbidity at the donor areas by decreasing the necessity of autogenous bone grafts and improved the patients' satisfaction and comfort. This case report aimed to evaluate the clinical and histological efficacy of deproteinized porcine bone mineral (DPBM) for socket preservation following molar extraction. A 48-year-old male patient underwent extraction of tooth #17 (maxillary left second molar), followed by socket preservation using DPBM. For comparative purposes, the patient's contralateral tooth #27 (maxillary right second molar) was extracted without socket preservation. After a 3-month follow-up, clinical evaluation revealed satisfactory dimensional stability of the alveolar ridge at the #17 site (bone height: 6.9 mm; bone width reduction: 7.4 mm), whereas the #27 site exhibited significant resorption (bone height: 6.4 mm; bone width: 6.9 mm). Histological analysis via Masson trichrome staining demonstrated new bone formation and tight integration between the DPBM particles and host bone at the #17 site. Subsequently, a dental implant was successfully placed at the preserved socket 4 months post-preservation, with stable osseointegration and functional restoration at the 1-year follow-up. This case confirms the effectiveness of DPBM in maintaining alveolar ridge dimensions and promoting new bone formation, providing a reliable foundation for subsequent implant therapy.

## Introduction

Tooth extraction often leads to alveolar ridge resorption, which can pose challenges for future dental prosthetic rehabilitation (1). In the first six months after tooth extraction, the loss of a horizontal bone and vertical bone attend to 29%–63% and 11%–22%, respectively (2). Alveolar ridge preservation techniques using bone graft materials aim to minimize this resorption and maintain the alveolar ridge's volume and architecture. Augmentation of the alveolar ridge using guided bone regeneration (GBR) became a treatment option to obtain bone support for osseointegrated dental implants (3). The procedures for bone augmentation of the alveolar ridge through GBR present successful, long-term follow-up results (4, 5).

Various biomaterials have been used as graft materials, such as autologous bone, bone substitutes (allografts, xenografts, and alloplasts), autologous blood products and bioactive substances (6–8). Autogenous bone grafts are often used in GBR procedures and are considered as the gold standard because it is the only biomaterial that combines properties of osteogenesis, osteoinduction, and osteoconduction (9, 10). However, disadvantages such as morbidity at the donor site, limited availability, tooth sensitivity, and risk of dehiscence of the wounds have led to investigations on the development and application of bone substitutes for the regeneration of the alveolar bone ridge (11, 12).

Deproteinized porcine bone mineral (DPBM) is a common intraoral augmentation biomaterial. However, detailed case reports with histological analysis, especially using Masson trichrome staining, are valuable for understanding the biological processes and long-term outcomes of the treatment (13). This case report presented a patient treated with DPBM for socket preservation, along with histological evaluation using Masson trichrome staining. The clinical examination was measured by Cone Beam Computed Tomography (CBCT) and subsequent restorations. The aim of the present case report was to explore the influences of DPBM on the socket preservation and its further outcomes for implant outcome.

## Case presentation

### Patient information

**Figure 1:**
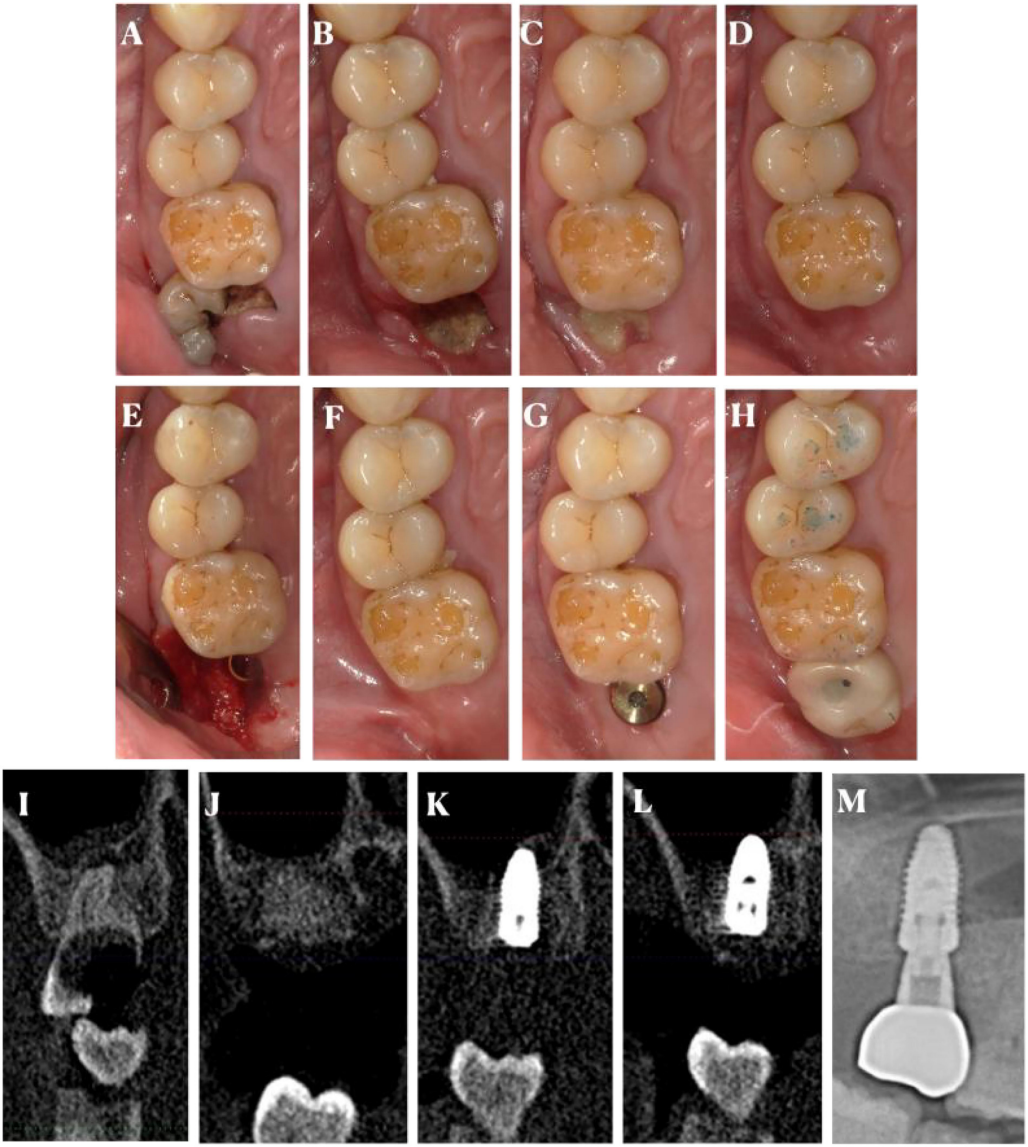
The procedures of the right side of #17. Intraoral photos **(A)**, before tooth extraction; **(B)**, after removed sutures; **(C)**, 1-month follow-up; **(D)**, before implant insertion; **(E)**, after implant insertion; **(F)**, before second stage operation; **(G)**, after second stage operation; **(H)**, after implant restoration; Radiograph (sagittal view of CBCT scan) **(I)**, before tooth extraction; **(J)**, before implant insertion; **(K)**, after implant insertion; **(L)**, before second stage operation; **(M)**, after implant restoration.

**Figure 2:**
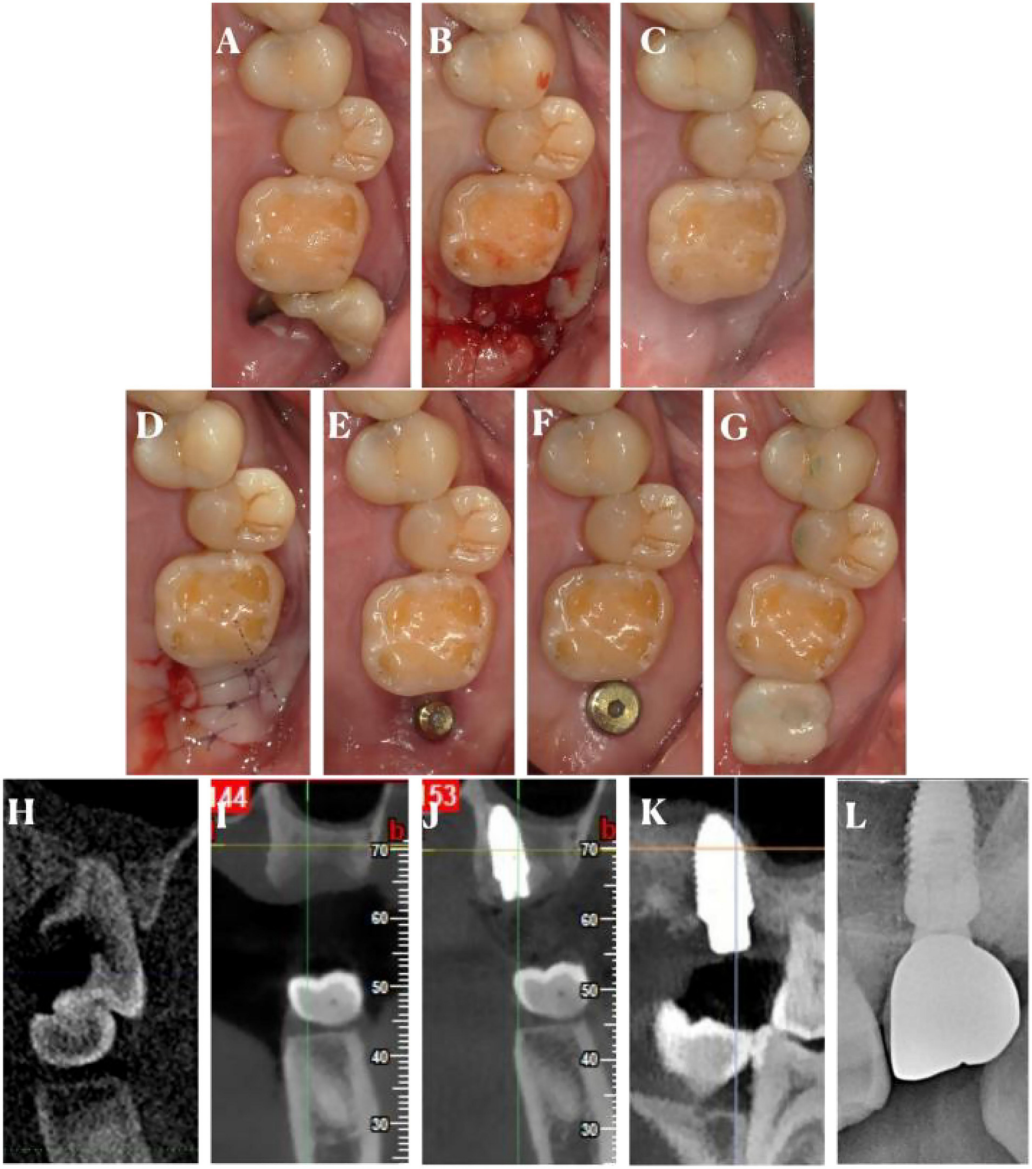
The procedures of the left side of #27. Intraoral photos **(A)**, before tooth extraction; **(B)**, after removed sutures; **(C)**, before implant insertion; **(D)**, after implant insertion; **(E)**, before second stage operation; **(F)**, after second stage operation; **(G)**, after implant restoration; Radiograph (sagittal view of CBCT scan) **(H)**, before tooth extraction; **(I)**, before implant insertion; **(J)**, after implant insertion; **(K)**, before second stage operation; **(L)**, after implant restoration.

A 48-year-old male patient presented to the dental clinic with two non-restorable maxillary second molar of #17 and #27 (Figures 1A, 2A). The patient referred to the Department of Oral at the Zhongshan Hospital Affiliated of Fudan University from June 2024 to October 2025. The patient had no systemic diseases, such as diabetes, cardiovascular diseases, or autoimmune disorders, with no history of smoking or significant drugs. The research was approved by the ethical committee of the Zhongshan Hospital Affiliated of Fudan University. (Number B2021-768R).

### Pre-operative evaluation

Clinical examination revealed a carious and severely damaged maxillary second molar with a deep periodontal pocket. Radiographic examination showed extensive bone loss around the tooth root, indicating the need for extraction. The alveolar bone height and width in the extraction site were measured on the CBCT images as a baseline for future comparison (Figures 1I, 2H).

### Tooth extraction and socket preservation procedure

Under local anesthesia, the maxillary second molar was carefully extracted using standard dental extraction techniques to minimize damage to the surrounding alveolar bone. After extraction, the socket was thoroughly cleaned of any debris, granulation tissue, and blood clots. DPBM (Purgo, Korea) was then gently packed into the extraction socket of #17 until it was completely filled. The graft material was carefully placed to ensure uniform distribution within the socket. The gingival tissue was then sutured to achieve primary closure. And another extraction socket of #27 was closed without bone graft.

### Post-operative management

The patient was prescribed antibiotics (amoxicillin 500 mg three times a day for 3 days) and non-steroidal anti-inflammatory drugs (ibuprofen 400 mg every 8 h as needed for pain) post-operatively. The patient was instructed to maintain strict oral hygiene, including gentle rinsing with a chlorhexidine-based mouthwash three times a day. The related data of pre-and post-operative CBCT values was shown (Table 1).

**Table 1:** The height and width of pre-and post-operative molar site via CBCT.

Molar site	The height of pre-extraction (unit: mm)	The width of pre-extraction (unit: mm)	The height of pre-implant (unit: mm)	The width of pre-implant (unit: mm)
Right site (17)	7.6	7.9	6.9	7.4
Left site (27)	7.4	7.8	6.4	6.9

### Follow-up

#### Clinical evaluation

At the 2-week post-operative visit, the sutures were removed, and the surgical site was examined (Figures 1B, 2B). There was no sign of infection, and the gingival tissue showed normal healing. At the 1-month follow-up, the gingiva was well-healed, and the extraction site was covered with healthy-looking soft tissue (Figure 1C). The alveolar ridge was palpated, and no significant resorption was clinically evident.

At the 3-month follow-up, a CBCT was taken to evaluate the alveolar ridge dimensions (Figures 1J, 2I). The results showed that the alveolar bone height and width had been well-maintained, with only a minimal reduction in comparison to the pre-operative measurements for the right site #17. For the left site #27, the bone reduction was more obvious. The reduction in bone height of #17 was less than 0.5 mm, and the reduction in bone width was approximately 0.5 mm, which was considered acceptable for future dental implant placement. Relatively, for the light site, the reduce in the bone height was 1 mm, along with 0.9 mm reduction in bone width.

The implants and restorations were placed in the molar site of #17 and #27 in Figures 1E–H, 2D–G. Furthermore, radiograph of #17 and #27 implant and restoration was shown in Figures 1K–M, 2J–L. Furthermore, we had reported outcomes of patient in the Table 2. The patient only has a different perception of pain and felt that #17 was more painful.

**Table 2:** Patient satisfaction after restoration placement.

Agreement percentage	The left extraction side (#17)	The right extraction side (#27)
Pain	9	8
Satisfaction	9	9
Healing quality	9	9
Chewing function	9	9
Dare to bite with dental implants (compared with your own teeth)	9	9
Sensation	9	9
Tooth color	9	9
Crown shape	9	9
Color of mucosa around the crown	9	9
Morphology of mucosa around the crown	9	9
Cleanliness	9	9
Comfort degree	9	9
Worthwhile spending money on dental implants	9	9

#### Histological evaluation

To further assess the bone regeneration and integration of the DPBM, a small biopsy was taken from the extraction site at the 3-month follow-up. The tissue sample was fixed in formalin, decalcified, and processed for histological analysis. Masson trichrome staining was performed to distinguish between different tissue components, such as collagen (blue), muscle (red), and nuclei.

**Figure 3:**
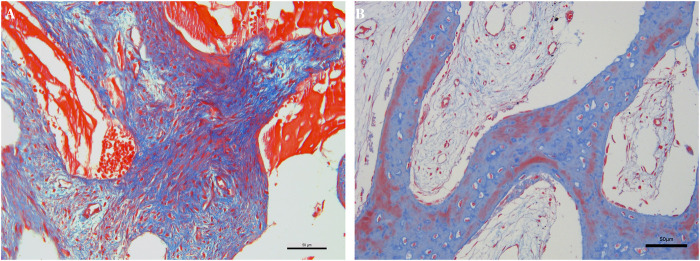
Masson trichrome staining. **(A)**, the staining of the right site of #17; **(B)**, the staining of the right site of #27.

The Masson-stained sections revealed new bone formation within the right extraction socket #17. The newly formed bone was characterized by the presence of osteocytes within lacunae and a well-organized collagen matrix, which stained blue. The DPBM were clearly visible and were surrounded by the newly formed bone tissue (Figure 3A). There was no sign of inflammation or foreign-body reaction around the graft particles. The collagen fibers in the connective tissue surrounding the graft and the newly formed bone were arranged in an organized manner, indicating proper tissue repair and remodeling. In contrast, the collagen fibers and new bone in the left extraction socket of #27 seemed less than that in right extraction socket of #17 (Figure 3B). There were noticeable areas of bone with a regular arrangement of collagen fibers, stained red, which may correspond to lamellar bone, indicating the completion of the bone remodeling process.

## Discussion

### Case analysis

This case demonstrated the effectiveness of DPBM in socket preservation. The minimal resorption of the alveolar ridge, as shown by the CBCT measurements, indicated that DPBM was able to maintain the integrity of the extraction socket and promote new bone formation. The histological analysis using Masson trichrome staining provided valuable insights into the biological processes occurring within the extraction site. The presence of new bone formation and the absence of inflammation or foreign-body reaction around the graft particles suggested that DPBM was well-tolerated by the host tissue and facilitated the regeneration of bone tissue. The “novel” DPBM with high biocompatibility is a natural mineral-based bone powder, and its key distinctions from existing porcine/bovine bone substitutes (e.g., Bio-oss) are systematically elaborated below, supported by relevant literature. Firstly, its morphological characteristics such us SEM (Scanning Electron Microscopy) and TEM (Transmission Electron Microscopy) images are similar to those of a commercial bovine bone (Bio-oss) (14). Secondly, It has a porosity of 72.4%, which is closer to that of human bone (76.5%) compared to bovine bone (63%—71%) (15). The high porosity of this graft enables it to absorb liquids more quickly than natural demineralized bovine bone matrix, resulting in a shorter post-operative healing waiting time for this graft compared to bovine bone. Thirdly, for sterilization methods, this deproteinized porcine bone mineral undergoes a heat treatment process at 1,300 ℃ and at a dose of 25 kGy for the inactivation of porcine virus which are different with Bio-Oss at a 300 ℃ heat treatment. It can get sufficient virus-reducing capacity to achieve a high margin of virus safety (16).

### Clinical significance

Socket preservation is crucial for the success of future dental implant placement and prosthetic rehabilitation. The use of DPBM, as demonstrated in this case, can effectively prevent excessive alveolar ridge resorption, providing a better foundation for dental implant placement. The histological evaluation using Masson trichrome staining not only confirmed the clinical findings but also enhanced our understanding of the bone-grafting process at the tissue level. This information can be used to optimize the alveolar ridge preservation techniques and improve the long-term outcomes of dental implant treatment.

### Comparison with previous studies

Previous studies have also reported the use of different bone graft materials for alveolar ridge preservation (17). Some studies have shown that autogenous bone grafts are the gold standard due to their osteogenic, osteoinductive, and osteoconductive properties. However, autogenous bone grafts have limitations, such as limited availability, donor-site morbidity, and increased surgical time (18, 19). Allogeneic and xenogeneic bone grafts, like DPBM, offer alternatives with good osteoconductive properties (20). Our case results are consistent with previous reports that have shown the effectiveness of DPBM-like bone grafts in maintaining alveolar ridge dimensions and promoting bone regeneration. However, more long-term and large-scale studies are needed to fully evaluate the performance of DPBM compared to other materials.

### Summary

In conclusion, this case report demonstrated the successful use of DPBM for alveolar ridge preservation following tooth extraction. The clinical and histological evaluations, especially the Masson trichrome staining analysis, provided evidence of the material's ability to maintain alveolar ridge dimensions and promote new bone formation. This case highlights the importance of alveolar ridge preservation techniques and the value of histological analysis in understanding the outcomes of bone-grafting procedures. Further research is needed to explore the long-term stability and optimal application of DPBM in different clinical scenarios.

## Data Availability

The original contributions presented in the study are included in the article/Supplementary Material, further inquiries can be directed to the corresponding author/s.
